# From the Editor’s Desk

**Published:** 2014-06

**Authors:** Paul A Brink

**Affiliations:** Department of Internal Medicine, Faculty of Health Sciences, University of Stellenbosch and Tygerberg Hospital, Tygerberg

Alas, this is my last time writing ‘From the Editor’s Desk’. The CVJA has a new editor-in-chief, Prof Patrick Commerford. He succeeds Prof AJ Brink, a founder of this journal and editor-in-chief until his death in October 2012. From the July/August issue, Prof Commerford will be responsible for overseeing the processing of articles through the editorial system, sourcing original articles where necessary, writing editorials, and maintaining the high standard of the journal.

CVJA is now in its 25th year, a quarter of a century old, and this event is soon to be celebrated. However, we certainly had a rocky ride after the death of Prof AJ Brink at 89, which, although not untimely, created an unanticipated void. We are certain Prof Commerford will create a smoother ride for all at the journal offices.

**Fig. 1.**
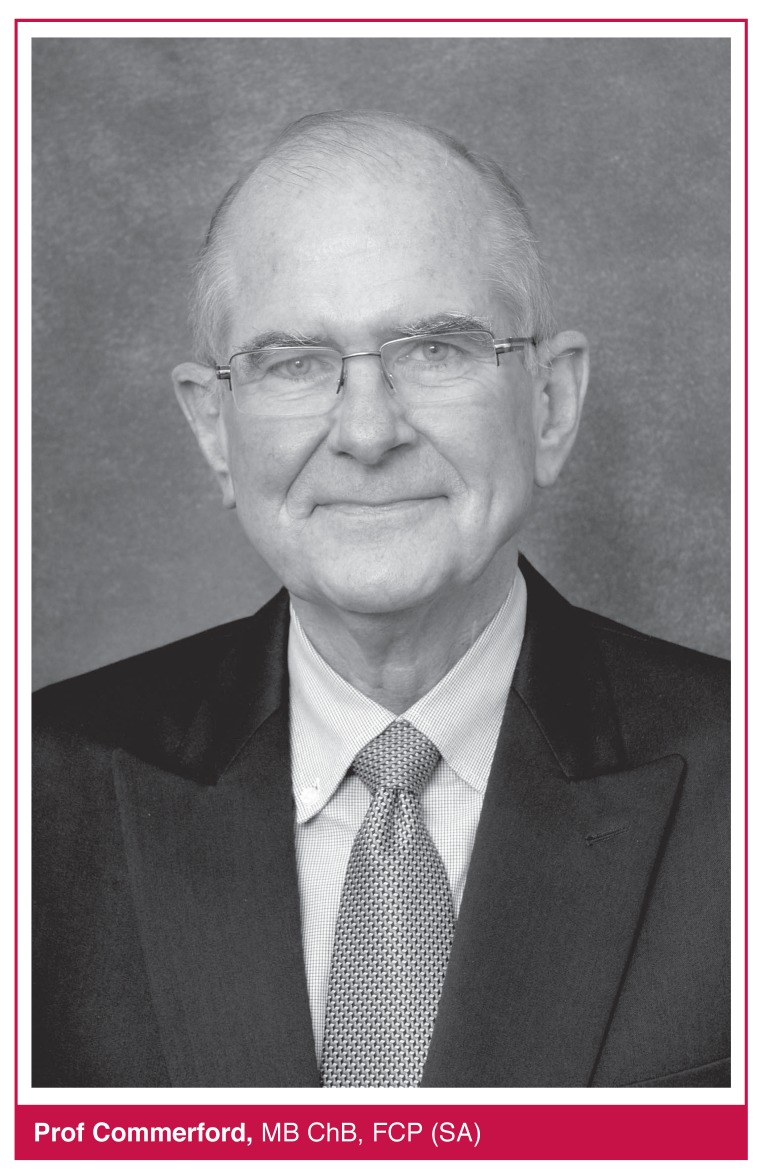
Prof Commerford, MB ChB, FCP (SA)

Prof Commerford (Fig. 1) is a well-respected clinician, educator, scientist, and an organiser and administrator. He has served on national medical and scientific bodies, even heading some. He has extensive experience in medical scientific writing and reviewing, and also, to the benefit of the journal, experience as an editor. I will remain intimately involved in the business side of the journal, maintaining it as a stable and financially viable venture.

Prof Commerford has served on the editorial boards of a number of journals, including CVJA. He recently retired as professor and head of the cardiology unit at Groote Schuur Hospital, where he occupied the Helen and Morris Mauerberger chair.

For this issue, we have as usual, reviews, original articles and case studies, the latter only available on the web. Scolch *et al.* touts the virtues of cardiac magnetic resonance (CMR) imaging. CMR is certainly impressive but one must remember that if the mountain cannot go to Mohamed, Mohamed must go to the mountain. In that regard, echocardiographic machines will not easily be replaced. They are cheaper and very mobile. You can take them to your patient in your rooms, the clinic, ward, ICU, theatre, and in distant places.

As a review article, Pop *et al.* (page 137) addresses nonstandard markers of cardiovascular risk in women, teasing out from other studies factors pertaining to woman.

Among the original studies, we have articles ranging from isolated perfused rat hearts and preconditioning in the basic science laboratory (Kelly-Laubscher *et al.*, page 118) to platelet morphology and myocardial perfusion in patients with diabetes mellitus from the clinical laboratory (Sarikaya *et al.*, page 110). From catastrophic carbon monoxide poisoning we learn of the effects on cardiac repolarisation (Eroglu *et al.*, page 106).

For the surgically minded, the safety of simultaneous coronary artery bypass grafting and carotid endarterectomy is described (Aydin *et al.*, page 130). There is an observational study on quality of life in patients with atherosclerosis obliterance versus those with Buerger’s disease (Karakoyun *et al.*, page 124).

We trust that you will enjoy this issue.

